# Stigma and its impact on Quality of Life among Early Career Mental Health Professionals

**DOI:** 10.1192/j.eurpsy.2024.165

**Published:** 2024-08-27

**Authors:** S. Marchini, C. Vidal Adroher, S. Alansari, B. Turan, A. Sulais, F. AlDandan, F. AlEid, M. Al Ali, R. Grujicic, A. AlMarshedi

**Affiliations:** ^1^Faculty of Medicine, Université Libre de Bruxelles (ULB); ^2^Psychiatry for Infant, Child, Adolescent and Youth, University Hospital Brussels (HUB), Brussels, Belgium; ^3^Psychiatry and Clinical Psychology, University of Navarra Clinic, Pamplona, Spain; ^4^Ministry of Community Development, Dubai, United Arab Emirates; ^5^Child and Adolescent Psychiatry, Karadeniz Technical University, Faculty of Medicine, Trabzon, Türkiye; ^6^Mental Health, King Fahad Specialist Hospital, Dammam; ^7^Psychiatry, King Fahad University Hospital (KFUH), Imam Abdulrahman bin Faisal University (IAU), Khobar, Saudi Arabia; ^8^Mental Health, Al Jalila Children’s Speciality Hospital, Dubai, United Arab Emirates; ^9^Clinical Department for Children and Adolescents, Institute of Mental Health, Belgrade, Serbia; ^10^Psychiatry, Prince Sultan Military Medical City, Riyadh, Saudi Arabia

## Abstract

**Introduction:**

Stigma towards mental health has been described as a major obstacle to seek help and access to mental health services. This could result in a worsened Quality of Life (QoL). There is a little evidence of stigma in Mental Health Professionals and its consequences, especially in Early Career ones (ECMPH), who can be a more vulnerable group. There is even more lack of studies with multicultural approaches. Exploring stigma, support systems and access to these, and the link of these factors with QoL is essential to develop effective strategies to support ECMHP, for both their own mental health and providing care to patients.

**Objectives:**

This study aims to explore the association between ECMHP’s stigma towards mental health and their QoL, and to identify predictors of QoL among this population.

**Methods:**

In this cross-sectional study, we designed an online survey to collect data among ECMHP, identified as having completed training since less than 7 years. QoL was assessed using the WHO-QoL. Stigma towards mental health was measured with the Opening Minds Stigma Scale for Health Care Providers (OMS-HC). Other general sociodemographic data were also collected. Descriptive results are resumed in absolute and relative frequencies for categorical variables. Student’s t-test and ANOVA were used to analyse scores in WHO-QoL and OMS-HC according to categorical variables. Pearson’s correlation coefficient was used to assess the association between WHO-QoL and OMS-HC. Simple and multiple linear regression were used to study the effect of stigma on QoL, taking into account potential confounders.

**Results:**

We collected data from 277 ECMHP from Europe (54.15%) and Asia (45.85%). Only 20% of our sample knew that their workplace has staff dedicated for mental health practitioners support, and among those, only 44% had visited it. OMS-HC total scores were significantly higher (***p<0,05*** *)* in nurses and practitioners without a sufficient support system and without a mental disorder. WHO-QoL total scores were significantly higher in participants with sufficient support systems, and without a mental or physical illness. There was a negative correlation between OMS-HC and WHO-QoL total scores. Univariate analysis showed that OMS-HC total scores predicted WHO-QoL total scores. In the multivariate analysis, OMS-HC total scores, having a mental illness and having sufficient support, independently predicted WHO-QoL total scores, even when adjusted for sociodemographic variables.

**Conclusions:**

Stigma towards mental health is related to QoL in ECMHP. Also, having sufficient support in the workplace improves QoL in this population. More studies are needed to help clarify the relationship between stigma and QoL using a longitudinal design.

**Disclosure of Interest:**

None Declared

